# Enrichment of Non-B-Form DNA at *D. melanogaster* Centromeres

**DOI:** 10.1093/gbe/evac054

**Published:** 2022-04-20

**Authors:** Venkata S.P. Patchigolla, Barbara G. Mellone

**Affiliations:** Department of Molecular and Cell Biology, University of Connecticut, Storrs, CT 06269, USA; Department of Molecular and Cell Biology, University of Connecticut, Storrs, CT 06269, USA; Institute for Systems Genomics, University of Connecticut, Storrs, CT 06269, USA

**Keywords:** centromere, satellite DNA, non-B DNA, centromere evolution, repetitive DNA, G-quadruplexes

## Abstract

Centromeres are essential chromosomal regions that mediate the accurate inheritance of genetic information during eukaryotic cell division. Despite their conserved function, centromeres do not contain conserved DNA sequences and are instead epigenetically marked by the presence of the centromere-specific histone H3 variant centromeric protein A. The functional contribution of centromeric DNA sequences to centromere identity remains elusive. Previous work found that dyad symmetries with a propensity to adopt noncanonical secondary DNA structures are enriched at the centromeres of several species. These findings lead to the proposal that noncanonical DNA structures may contribute to centromere specification. Here, we analyze the predicted secondary structures of the recently identified centromere DNA sequences of *Drosophila melanogaster*. Although dyad symmetries are only enriched on the Y centromere, we find that other types of noncanonical DNA structures, including melted DNA and G-quadruplexes, are common features of all *D. melanogaster* centromeres. Our work is consistent with previous models suggesting that noncanonical DNA secondary structures may be conserved features of centromeres with possible implications for centromere specification.

**Significance**

Centromeres are essential genetic loci that mediate accurate chromosome segregation during cell division. How centromeres are specified is not fully understood. Although the role of a specialized type of chromatin in marking the centromere is clear, the significance of centromeric DNA in this process is still elusive. In this work, we show that the centromeric DNA of *Drosophila melanogaster* has a propensity to form noncanonical secondary DNA structures, supporting the hypothesis that these unconventional DNA conformations may be conserved and may contribute to centromere specification.

## Introduction

Eukaryotes share a common mechanism to faithfully segregate genetic information during each cell cycle by which chromosomes are attached to microtubule fibers and are physically pulled toward opposite poles by the kinetochores. Centromeres are essential chromosomal regions that specify the site for the assembly of the kinetochore and are epigenetically marked by chromatin enriched in the histone H3 variant centromeric protein A (CENP-A). CENP-A has been shown to be sufficient for kinetochore assembly and de novo recruitment of CENP-A in *Drosophila melanogaster* somatic cells (Mendiburo et al. 2011; Chen et al. 2014; Palladino et al. 2020). Despite their conserved and essential function, centromeres are among the most rapidly evolving regions of genomes (Melters et al. 2013). This rapid evolution has been proposed to be a result of intragenomic conflict whereby centromeres act as selfish genetic elements driving the rapid evolution of centromeric proteins (Henikoff et al. 2001; Malik and Henikoff 2009). Centromeres typically form on highly repetitive DNA often interspersed with transposable elements (reviewed in Mellone and Fachinetti 2021). In organisms such as fungi, nematodes, insects, plants, and vertebrates, centromere function is largely independent of the presence of centromeric DNA sequences, relying instead on the presence of CENP-A chromatin (reviewed in McKinley and Cheeseman 2016 and Mellone and Fachinetti 2021). Thus, for most species, the functional significance of centromeric DNA sequences in dictating (or at least contributing to) centromere identity remains unclear.

In an effort to identify genetic characteristics shared among the centromeres of diverse eukaryotes, Kasinathan and Henikoff (2017) surveyed centromeric DNA sequences from mouse, chicken, *Schizosaccharomyces pombe*, and humans for the presence of <10-bp dyad symmetries (a.k.a. inverted repeats), which are known to adopt unconventional secondary structures such as stem-loops or cruciform extrusions. The authors found that the centromeres of species such as the African Green monkey, chicken, and the fission yeast *S. pombe* were enriched in these motifs. Centromeres enriched in dyad symmetries also showed a predicted propensity to form noncanonical secondary DNA structure under stress, such as that resulting from DNA supercoiling caused by transcription or replication. Noncanonical DNA structures are known as non-B-form DNA and collectively represent any deviation from double-stranded B-DNA (the right-handed helix with 10-nt per turn). The high likelihood of predicted cruciforms correlated with enrichment in dyad symmetries and other noncanonical DNA structures, such as melted DNA, were also predicted for some species. Interestingly, centromeres devoid of dyad symmetries, such as those of humans, contain binding sites for CENP-B, a protein that binds specifically to CENP-B box DNA motifs found within α-satellite (Verdaasdonk and Bloom 2011). CENP-B binding results in the bending of DNA (Tanaka et al. 2001), which in itself represents another noncanonical DNA structure. Based on these analyses, the authors proposed that noncanonical secondary structures may have been selected during centromere evolution, with a possible role as a structural cue for centromere specification (Kasinathan and Henikoff 2017). Consistent with this model, various non-B structures such as hairpins (Jonstrup et al. 2008, Chardon et al. 2022) and R-loops (Kabeche et al. 2018) have been observed at centromeres in vitro and in vivo. Oligos for *Drosophila*’s *dodeca* repeat (present only on centromere 3) and a 17 bp segment of human’s α-satellite, both of which are centromeric, formed i-motifs in vitro, however, these were only stable in acidic conditions (Garavís, Escaja, et al. 2015, Garavís, Mendez-Lago, et al. 2015). How widespread centromeric non-B-DNA structures are across species remains unknown.

**Fig. 1.**
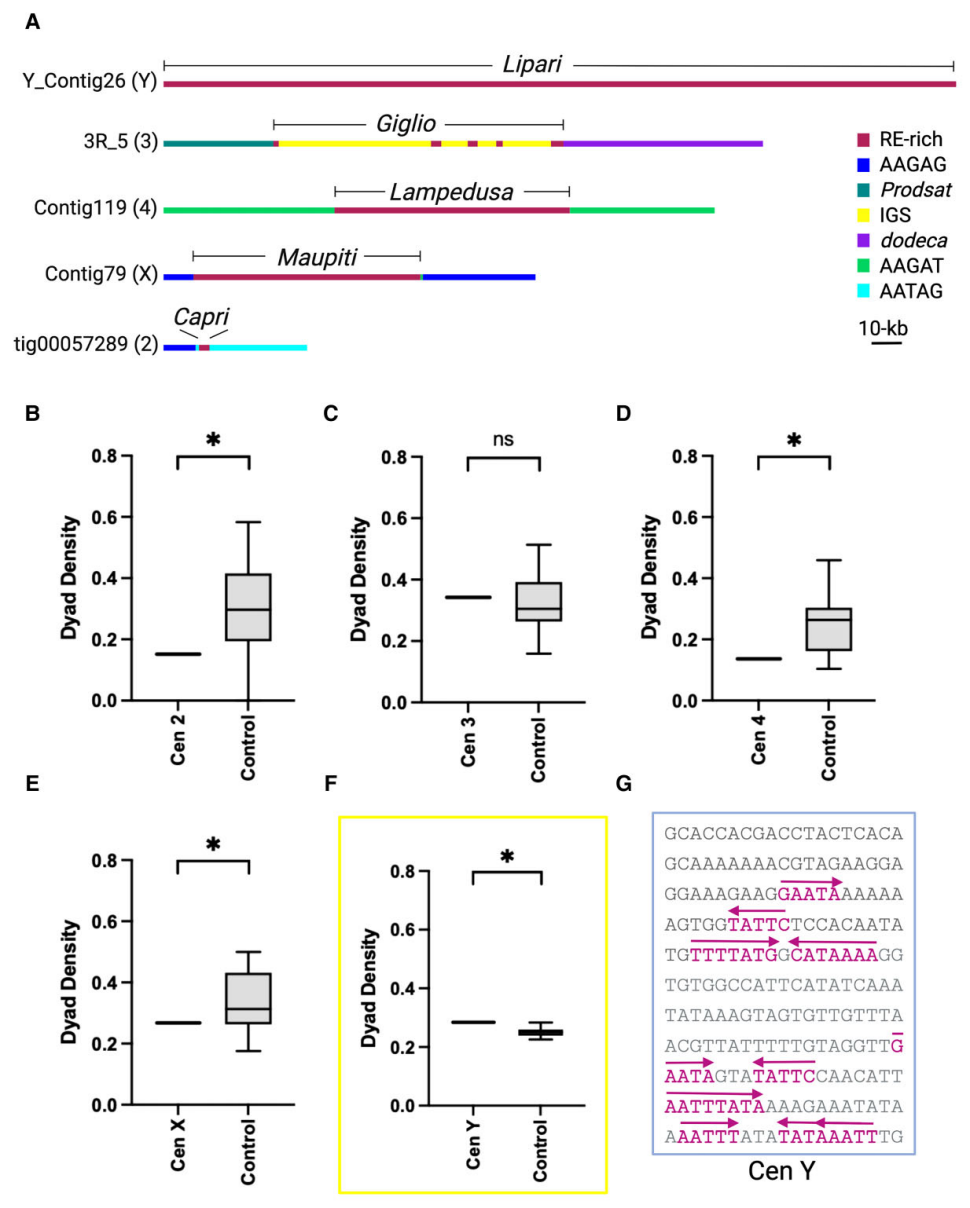
Dyad symmetries are not common features of *D. melanogaster* centromeres. (*A*) Simplified schematic of the DNA organization of *D. melanogaster* centromere contigs derived from Chang et al. (2019). Labels indicate the span of the islands of complex repeats enriched in retroelements (RE-rich). The centromere 3 island (*Giglio*) also contains copies of the ribosomal intergenic spacer (IGS). The remaining repeats (*dodeca*, *Prodsat*, etc.) are simple satellites flanking the islands. Note that CENP-A is associated with both the islands and the flanking satellites shown. (*B*–*F*) Box-and-whisker plots of dyad symmetry densities for *D. melanogaster* centromeres. The two whiskers on either side of the box represent the 1st and 4th quartile of the data, whereas the two inner boxes, separated by the central line (the median) represent the 2nd and 3rd quartile of the data. Only the Y contig (Y_Contig26; highlighted by box) showed a significant enrichment. *P* < 0.05, one-sample *t*-test. (*G*) Example of inverted repeats from the Y centromere contig (base pairs 181–390).

The centromeres of *D. melanogaster* were identified recently through a combination of long-read sequencing, chromatin immunoprecipitation, and OligoPaints Fluorescence In-Situ Hybridization (FISH). Chang et al. (2019) identified five contigs that make up at least part of the centromeres for the five the *D. melanogaster* chromosomes (X, 2, 3, 4, and Y) (fig. 1*A*). The contigs for centromeres X, 3, and 4 are composed of an island of complex DNA enriched in retroelements flanked by simple satellite repeats. For centromere 2, only a short contig was identified, which contains a small island with a single truncated retroelement flaked by simple satellites. Lastly, the contig for the Y centromere consists of a large island and no satellite DNA. Although commonly centromeres are made up of specific repeats, none of the repeats found in *D. melanogaster* centromeric contigs are unique to the centromeres, even though they display unique arrangements and are enriched at centromeres. For centromeres X, 2, and 4, the CENP-A domain spans a region larger than the contig themselves, which, based on cytological analyses, can be inferred to be made up of unassembled simple satellites. Importantly, FISH combined with immunofluorescence on extended chromatin fibers showed that ∼70% of the CENP-A domain sits on the island, whereas the remaining 30% is associated with the flanking satellites. Thus, both islands and flanking satellites are components of the “active” centromere (i.e., the CENP-A-rich region is where the kinetochore forms) (Chang et al. 2019). The remaining flanking satellites, not CENP-A associated, are presumed to be heterochromatic and to form the pericentromere.

Here, we use several prediction algorithms to survey the presence of non-B-DNA-form at the centromeres of *D. melanogaster.* Although we show that inverted repeats and cruciform extrusions are not a predominant feature at *D. melanogaster* centromeres, we find evidence for the enrichment of other predicted noncanonical secondary structures such as melted DNA and G-quadruplexes.

## Results and Discussion

### Dyad Symmetries are Not Common Features of *D. melanogaster* Centromeres

To determine if *D. melanogaster* centromeres are enriched in <10-bp DNA dyad symmetries as previously reported for the centromeres of other species (Kasinathan and Henikoff 2017), we used the program Palindrome from the EMBOSS suite. We used five contigs (one for each of the X, 2, 3, 4, and Y chromosomes) that are highly enriched in CENP-A chromatin immunoprecipitations and were confirmed to be associated with CENP-A using OligoPaint FISH on extended chromatin fibers as the bona fide *D. melanogaster* centromeres (Chang et al. 2019) (fig. 1*A*) for our analyses. We refer to these contigs as “centromeres” throughout this paper. For our controls, we used several composition and length-matched random genomic sequences for each of the centromere contigs (see Materials and Methods). We used the EMBOSS palindrome output to calculate dyad densities obtained by adding the number of base pairs that are part of a dyad divided by the sequence length and plotted these using box-and-whisker plots. We find that only the Y centromere displays dyad symmetry densities higher than control average (fig. 1*B*–*G*). These analyses suggest that dyad symmetries are not major features of *D. melanogaster* centromeres and thus are unlikely to play a role in centromere specification in this species. A lack of dyad symmetries was previously reported for human, great apes, and *M. musculus* centromeres (Kasinathan and Henikoff 2017).

### Enrichment of Predicted Non-B-Form DNA Structures at Centromeric Contigs Using SIST

The EMBOSS palindrome algorithm identifies dyad symmetries based on sequence analysis. However, this algorithm does not take into account the predicted thermodynamics of DNA and thus does not provide information on the secondary structures it is likely to adopt. Superhelical transitions occur in DNA when negative supercoiling drives susceptible regions to acquire forms alternative to native B-DNA that are energetically favorable. To determine if centromeres are susceptible to adopt non-B-form DNA, we used a computational algorithm that models stress-induced structural transitions (SIST) for multiple noncanonical DNA secondary structures: Z-DNA, DNA melting (i.e., strand separation), and cruciform extrusions (Zhabinskaya et al. 2015). SIST was previously used by Kasinathan and Henikoff (2017) to show higher probability to adopt non-B-form DNA for centromeres enriched in dyad symmetries.

**Fig. 2.**
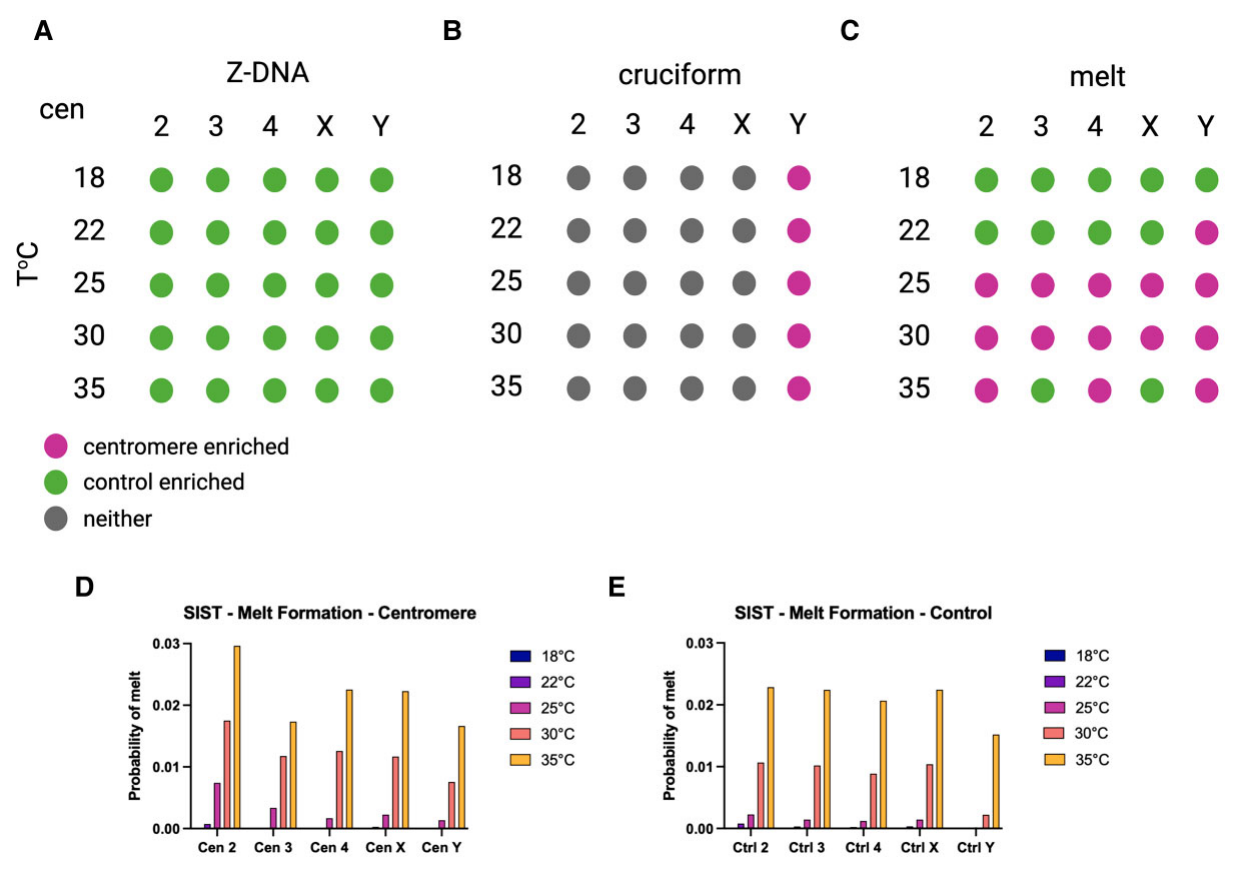
Enrichment of predicted non-B-form DNA at centromere contigs using SIST. Diagram summarizing the SIST outputs. Results for Z-DNA (*A*), cruciform (*B*), and melted DNA (melt) (*C*) are shown for each of the centromeres at five different temperatures (°C). Enrichment of non-B DNA in controls suggests depletion of these forms at centromeres. Different colors represent significance as outlined in the legend. (*D*) Average probability of melted DNA for each centromeric contig identified by SIST. The bars represent the average probability of formation for melted DNA at a given temperature and centromere. (*E*) Average probability of melted DNA for each control identified by SIST. The bars represent the average probability of formation for melted DNA at a given temperature and set of controls.

We ran segments of DNA in 5,000-bp blocks every 2,500-bp and took the maximum values for the overlapping regions whenever different. DNA transitions depend on temperature; because *D. melanogaster* is an ectotherm species, we ran SIST at five different temperatures at which *D. melanogaster* may be found (18, 22, 25, 30, and 35 °C) and determined enrichment probabilities for centromeres compared with their respective control regions. The probability of Z-DNA formation, which has not been previously analyzed for centromeres, is lower than controls for each of the centromeres irrespectively of the temperature suggesting that centromeres are depleted of Z-DNA compared with control regions of the genome (fig. 2*A*). As for cruciforms, only the centromere of the Y chromosome shows higher probability than controls at all temperatures (fig. 2*B*). These findings are consistent with the observation that the Y is the only centromere showing an enrichment of inverted repeats (fig. 1*F*), which are thought to adopt cruciform extrusions (Hamer and Thomas 1974; Leach 1994).

Interestingly, at the more physiologically relevant temperatures of 25 and 30 °C, all of the centromeres display higher probability than controls for melted DNA (melt), another DNA form considered noncanonical (Kasinathan and Henikoff 2017). The Y displays higher DNA melting probability than controls at all temperatures >22 °C. At 18 °C, none of the centromeres displays higher probability of DNA melting (fig. 2*C*). Although the probabilities of melted DNA continue to increase with increasing temperatures for centromeres 3 and X, the probabilities for the controls increase more (fig. 2*D* and *E*), resulting in the controls being enriched at 30 and 35 °C (fig. 2*C*).

Our findings in *Drosophila* are consistent with previous analyses on the centromeres of fission yeast, African green monkey, and on human neocentromeres, where the probability of melted DNA was found to be higher than that of controls (Kasinathan and Henikoff 2017). Cells’ and organisms’ growth are regulated by temperature and the temperatures at which different organisms thrive are vastly different across eukaryotic species. Given that the ability of centromeres to adopt non-B DNA conformations needed for proper centromere function during cell division is also affected by temperature, this could be a factor under selection during evolution, contributing to the diversity of centromeric DNA sequences observed across lineages.

DNA melting is accurately predicted at actively transcribed regions that display strand separation in vivo (Zhabinskaya et al. 2015). As centromeres from across species have been shown to display transcriptional activity (reviewed in Mellone and Fachinetti 2021), the enrichment for this particular noncanonical DNA structure is especially interesting. DNA melting may facilitate transcription, which in turn could facilitate histone turnover or the formation of secondary DNA/RNA structures at centromeres, contributing to centromere specification (Kasinathan and Henikoff 2017; Talbert and Henikoff 2020; Mellone and Fachinetti 2021).

### Enrichment of Non-B-Form DNA in Centromeric Contigs Using GQuad

**Fig. 3.**
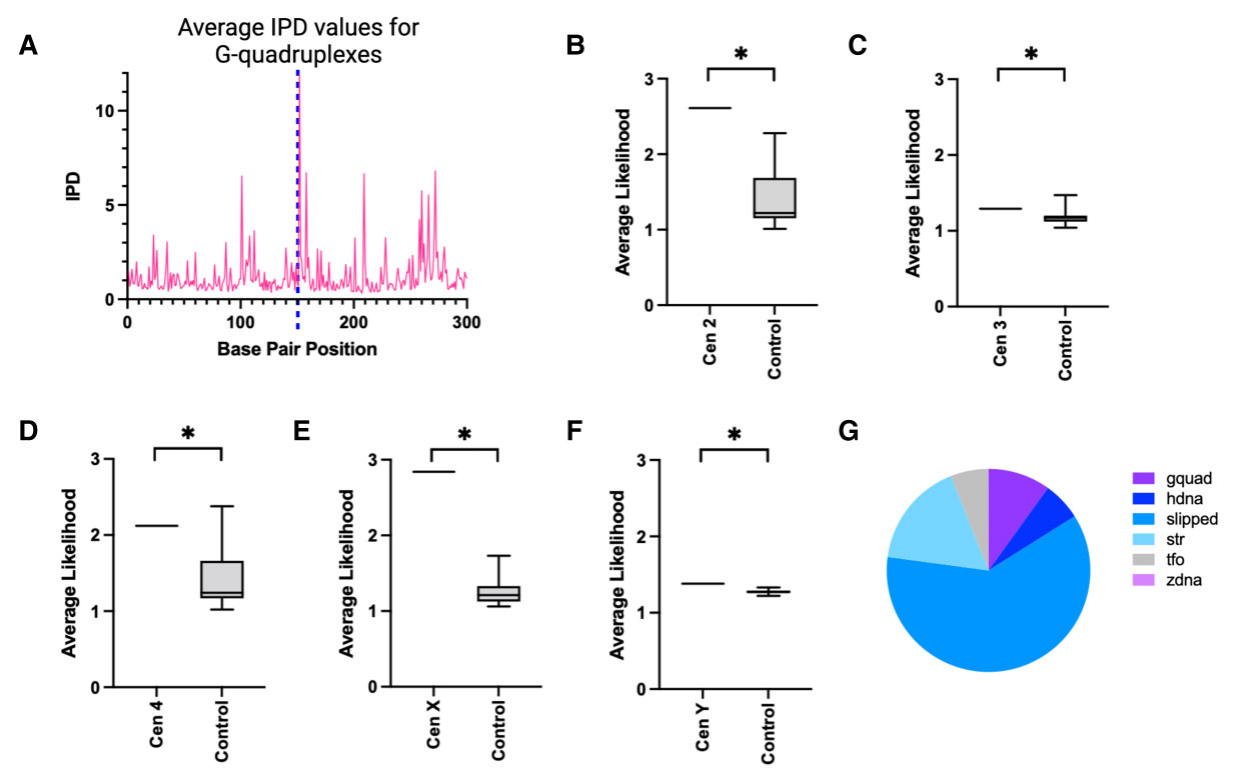
Enrichment of predicted non-B-form DNA in centromeric contigs using GQuad. (*A*) Plot showing the average IPD value for sequences predicted to form G-quadruplexes by GQuad with a likelihood of two asterisks (see text for details). G-quadruplexes are centered around 150-bp. (*B*–*F*) Box-and-whisker plots of the data distribution of likelihoods for each of the centromeres as a combination of all non-B DNA predicted by Gquad. The two whiskers on either side of the box represent the 1st and 4th quartile of the data, whereas the two inner boxes, separated by the central line (the median) represent the 2nd and 3rd quartile of the data. Asterisks represent *P* < 0.05 (KS test). (*G*) Pie chart showing the relative contributions of different non-B DNA types identified by Gquad. See also supplementary figure S1, Supplementary Material online.

Previous work proposed that non-B-form DNA may be an evolutionary conserved signature required for centromere specification. Yet, aside from the Y centromere, which is enriched in inverted repeats and has higher probability of forming cruciforms than controls (figs. 1*F* and 2*B*), all other *D. melanogaster* centromeres show higher probability than controls only for DNA melting. As SIST only predicts three types of noncanonical DNA structures, we wanted to expand our analysis to additional non-B-form DNA types. For this purpose, we used Gquad, a package that can predict seven different non-B DNA structures: a-phased DNA repeats, G-quadruplexes, intramolecular triplexes (H-DNA), slipped DNA, short tandem repeats (STR), triplex forming oligonucleotides (TFO), and Z-DNA. Gquad provides the positions and probability for specific non-B-form DNA using scores ranging from one asterisk (low likelihood) to three asterisks (high likelihood). In the absence of experimental data identifying non-B-form DNA and of a non-B-form DNA database for *D. melanogaster*, sequences known to form non-B-form DNA are not available as positive controls to determine the accuracy of our predictions. A previous study used interpulse duration (IPD) values (i.e., the time it takes to add a nucleotide during single-molecule sequencing) from PacBio long-read sequencing data to infer non-B-form DNA (Guiblet et al. 2018). When we plotted the average IPD values of regions predicted to form non-B-DNA (e.g., G-quadruplexes) identified by Gquad with a likelihood of two asterisks in a 300-bp window centered on the sequence predicted to form G-quadruplexes, we observed IPD values that were over twice as high, suggesting that the predictions generated by Gquad are likely to be accurate (fig. 3*A*). Next, we calculated all the likelihoods for each type of non-B-DNA and combined them such that if a particular base pair was predicted to form non-B-form DNA of more than one type, the likeliness of the two was added together. To determine the significance of enrichment we used the two-sample Kolmogorov–Smirnov (KS) test. Through this analysis, we find that all centromeres are significantly enriched for non-B-DNA (fig. 3*B*–*F*). Because the values for the seven types of non-B-DNA are combined in this analysis, we next wanted to determine which types of non-B-DNA are contributing most to the enrichment of non-B form DNA at the centromeres found with Gquad. For this, we analyzed the enrichment of individual types and found that of the seven noncanonical DNA forms, the ones that contribute the most are slipped DNA, STR, and G-quadruplexes (fig. 3*G* and supplementary fig. S1, Supplementary Material online). Similarly to melted DNA, slipped DNA forms when complementary DNA strands denature with the difference that in slipped DNA, direct repeats can reanneal in a mis-paired fashion with a potential to cause repeat expansion during replication (Sinden et al. 2007). STRs are common in highly repetitive DNA, whereas G-quadruplexes consist of single-stranded DNA rich in repeated guanines that fold forming stacked planar quartets (Lightfoot et al. 2019).

Next, we sought to determine which types of repeats are contributing most to the likelihood of adopting noncanonical DNA secondary structures by ranking the average Gquad values for all repeats in the *D. melanogaster* genome. We find that simple satellite DNAs contribute the most, as they are consistently ranked higher than other elements (supplementary table S1, Supplementary Material online). Short satellites are known to be prone to form noncanonical DNA structures, particularly slipped DNA (Sinden et al. 2007). If centromeres need to be marked by unconventional DNA structures in order to function or be stable, a potential explanation for why satellite DNA is found at many regional centromeres across species could be that it can adopt non-B DNA.

To determine the prevalence of non-B-DNA at centromeric contigs compared with the rest of the genome (irrespective of GC content), we ranked all contigs that make up the genome based on the average Gquad likelihood. We find that all centromeric contigs fall within the top 37% of the 190 contigs, with centromeres X, 2, and 4 ranking 6th, 15th, and 22nd, respectively (supplementary table S2, Supplementary Material online). These findings indicate that, although the centromeres may not rank the highest, they are among the most likely sequences in the genome to form non-B-DNA.

To determine the relative contribution of the islands versus the flanking satellite to the probability of adopting non-B DNA, we generated cumulative plots across each centromere contig for both the SIST probabilities (supplementary fig. S2, Supplementary Material online) and for Gquad likelihoods (supplementary fig. S3, Supplementary Material online) because these algorithms predict distinct types of non-B-DNA. We found that although SIST shows higher probability for non-B DNA on the islands, Gquad shows higher likelihood for non-B DNA on the satellites (supplementary fig. S1, Supplementary Material online). These findings suggest that both islands and flanking satellites can adopt different types of non-B DNA, consistent with the fact that CENP-A is associated with both types of repeats.

### G-quadruplexes are Common Features of *D. melanogaster* Centromeres

**Fig. 4.**
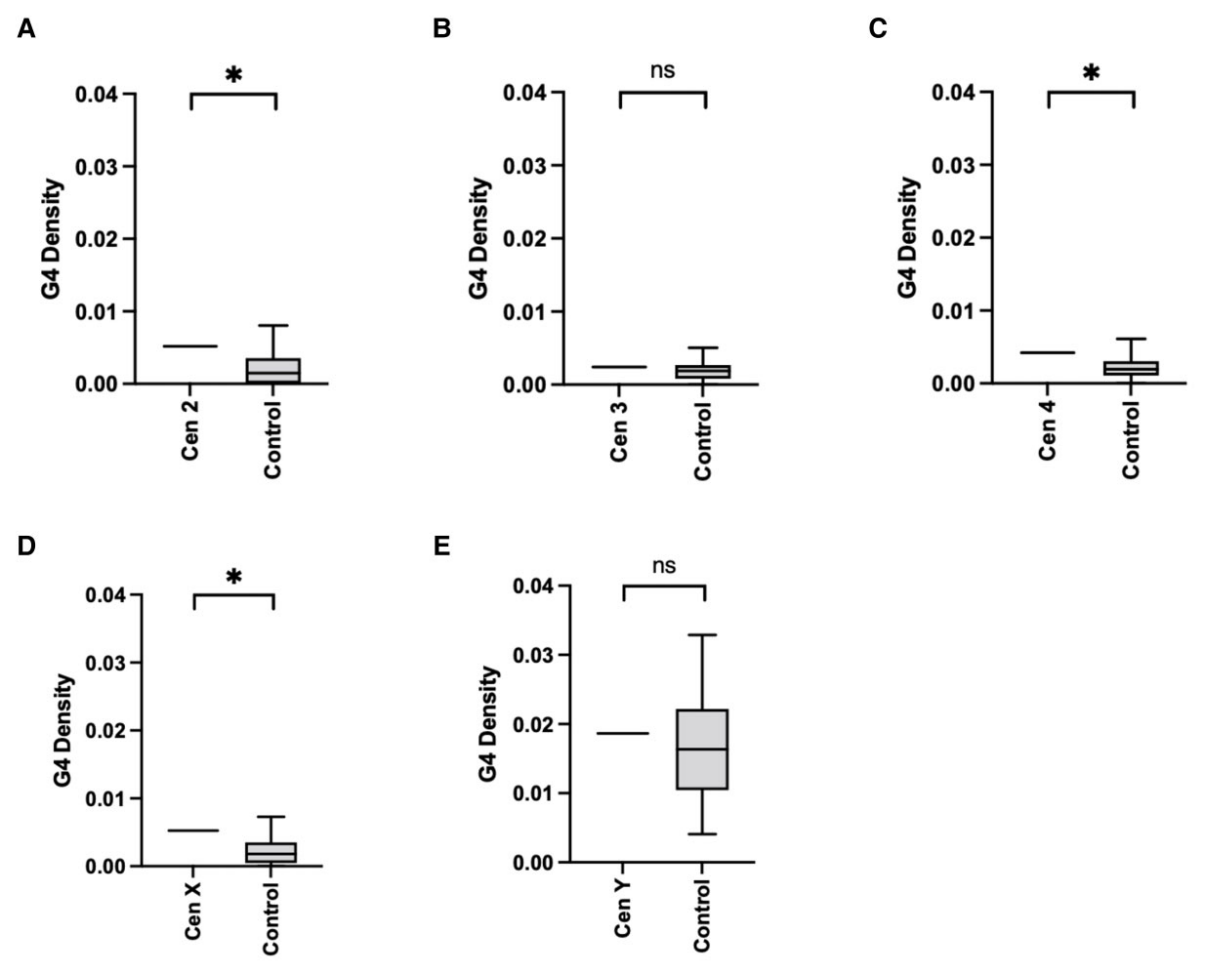
G-quadruplexes are common predicted features of *D. melanogaster* centromeres. (*A*–*E*) Box-and-whisker plots of the average G-quadruplex density for each centromere contig predicted by G4Hunter. The two whiskers on either side of the box represent the 1st and 4th quartile of the data, whereas the two inner boxes, separated by the central line (the median) represent the 2nd and 3rd quartile of the data. Asterisks represent *P* < 0.05 (one-sample *t*-test). Note that several control regions were not predicted to form any G-quadruplexes.

To confirm our prediction of G-quadruplexes at the centromeres with an additional algorithm, we used G4Hunter, a more recent program that gives a G-quadruplex propensity score as output. Unlike Gquad, G4hunter takes into account G-richness and G-skewness of a given sequence. Furthermore, this algorithm was validated on published sequences known to form G-quadruplexes as well as with biophysical methods (Bedrat et al. 2016). We ran G4Hunter using a stringent threshold value of 1.5 and found that all centromeres, except the 3 and Y centromeres, are enriched in G-quadruplexes compared with their respective controls (fig. 4*A*–*E*). Having observed enrichment of G-quadruplexes with two independent methods, we conclude that G-quadruplexes are likely to be common features of *D. melanogaster* centromeres. G-quadruplexes play a role in transcriptional regulation, translation, and replication (Bedrat et al. 2016). One possibility is that the higher prevalence of G-quadruplexes at the centromeres may contribute to centromere transcription homeostasis.

Collectively, our computational predictions suggest that *D. melanogaster* centromeres are enriched in non-B DNA. In particular, we observe enrichment of a subset of noncanonical secondary structures such as G-quadruplexes, melted DNA, and slipped DNA. Although none of the algorithms we used in our analyses include i-motifs predictions, it is possible that centromeric sequences also adopt this structure, as previously suggested by in vitro experiments with the *dodeca* satellite (Garavís, Mendez-Lago, et al. 2015). G-quadruplexes have been implicated in transcriptional regulation (Du et al. 2008) and transcription may be facilitated in regions of melted DNA; therefore, these structures could modulate transcriptional activity at centromeres. Furthermore, noncanonical DNA structures could be recognized by centromeric proteins with DNA binding properties. For example, HJURP, the chaperone that deposits CENP-A in tetrapods, recognizes cruciform structures known as Holliday junctions (Kato et al. 2007; Kasinathan and Henikoff 2017).

Similarly to *D. melanogaster* centromeric/pericentromeric satellites, human α-satellite DNA does not contain dyad symmetries. However, α-satellite harbors a 17-bp binding motif for CENP-B, which induces kinks and loops on this DNA (Tanaka et al. 2001; Chardon et al. 2022) that constitute noncanonical secondary structures. Although CENP-B related genes have been found in *Drosophila*, it is unclear whether or not they bind to the centromeres (Mateo and González 2014). A few satellite-binding proteins that may or may not alter the structure of DNA have been identified, such as the AATAT*_n_*-binding protein D1 (Aulner et al. 2002) and the *Prodsat* (AATAACATAG*_n_*)-binding protein Prod (Torok et al. 1997). However, whether or not these or other satellite-binding proteins occupy the CENP-A-associated portion of satellites is unknown.

The strength of our study is that it analyzes individual centromeres, revealing differences between them. The most striking difference we observed among them is that the Y centromere is the only one enriched in dyad symmetries with high probability of cruciform formation. Interestingly, the Y centromere is also the only one not containing CENP-A associated flanking simple satellites, which our analyses suggest are contributor to non-B DNA formation. Perhaps the lack of satellites at this centromere resulted in the selection for cruciform-forming repeats.

Our findings are consistent with the model that noncanonical DNA forms may be evolutionarily conserved features of centromeres with possible functions in centromere specification. Under such paradigm, the only feature under selection at centromeres would be their secondary DNA structure. Because a myriad of primary DNA sequence combinations can accommodate noncanonical secondary DNA conformations, such mechanism for centromere specification would enable ample opportunity for adaptation under intragenomic conflict (Kasinathan and Henikoff 2017).

## Materials and Methods

### Genome Data

The *D. melanogaster* genome used in this paper is from Chang and Larracuente (2019). The centromere contigs used for this analysis were Contig79 for centromere X, Contig119 for centromere 4, Y_Contig26 for centromere Y, Contig 3R_5 for centromere 3, and tig00057289 for centromere 2 (Chang et al. 2019).

### Source Code

Code used to perform the analysis in this manuscript is available on GitHub (https://github.com/venkata14/dmel-nonb).

### Generation of Controls Regions

The controls used for the analysis were 50 random segments of the genome that are both the same size and have a similar GC content within 10% as the respective centromeric contig. A maximum of two controls with a 50,000-bp overlap was allowed. A list of all the coordinates for the controls can be found on GitHub (https://github.com/venkata14/dmel-nonb).

### Detection of Dyad Symmetries Using EMBOSS Palindrome

EMBOSS Palindrome (https://www.bioinformatics.nl/cgi-bin/emboss/help/palindrome) was used to detect dyad symmetries with the minimum palindrome being 5, the maximum palindrome being 100, allowing a gap limit of 20 and allowing overlapping dyad symmetries. We analyzed the output by calculating the dyad density, which we defined as the sum of the lengths of all palindromic regions identified by Palindrome divided by the length of the entire contig containing it that contain that position. For a sequence, the length-normalized dyad density was defined as the sum of the values for each position divided by the sequence length.

### Prediction of Z-DNA, DNA Melting, and Cruciform Transitions Using SIST

The probabilities of Z-DNA, Cruciform transitions, and DNA melting were predicted using SIST (Zhabinskaya et al 2015) as described in Kasinathan and Henikoff (2017). We used default parameters with the algorithm type “A” which uses the trans_compete C++ codes along with five different temperatures: 18, 22, 25, 30, 35 for this analysis. For sequences >10 kb in length, we slid a 5,000-bp window in 2,500-bp steps and analyzed these subsequences using SIST. The SIST predictions were then reassembled by taking the maximum SIST value for any given base pair.

To determine the average probability of non-B-DNA formation for each temperature for all centromeres, we added the average value of Z-DNA, cruciform, and melt formation at each temperature.

### Prediction of Non-B-DNA Using Gquad

Gquad (v2.2-1; https://cran.r-project.org/web/packages/gquad/gquad.pdf) consists of multiple R packages that predict individual forms of non-B-DNA. We ran R packages on the heterochromatin-enriched *D. melanogaster* genome (Chang and Larracuente 2019) for the seven types of non-B-DNA: aphased DNA, G-quadruplexes, H-DNA, slipped DNA, STR, TFO, and Z-DNA. The packages output likelihoods for each nucleotide from a range of one to three asterisks representing the likelihood of non-B-DNA formations. For those that did not output a likelihood, we used two asterisks as the default likelihood value. We then analyzed the data by combining all likelihoods for the seven types of non-B-DNA for a respective sequence such that if there were overlaps in likelihoods of two different non-B-DNA types, we added those likelihoods together. This results in an array where each position is a summation of all likelihoods for a particular base pair.

### Identifying Relative Amounts of Non-B-DNA Using Gquad

Using the Gquad R package, we ran the package on the heterochromatin-enriched *D. melanogaster* genome (Chang and Larracuente 2019) for the seven types of non-B-DNA as similar to above. We then added all the positions predicted to form non-B-DNA for each of the seven types and created a pie chart. To determine significance of prevalence between specific types of non-B-DNA in the centromere versus the controls, we used the one-sample *t*-test on the average centromeric value and the control values for each respective non-B-DNA.

### Prediction of G-Quadraplexes Using G4Hunter

G4Hunter (https://www.bioinformatics.nl/cgi-bin/emboss/help/palindrome) was run using a window size of 25 base pairs and threshold values of 1 and 1.5. The program outputs the positions of the nucleotides that are predicted to form G-Quadruplexes. Using these positions, we calculated the density of G-Quadraplexes by taking the total number of nucleotides predicted to form G-Quadraplexes and dividing them by the total number of nucleotides in the respective sequence.

### Validating Non-B-DNA Predictions of Gquad Using IPDs

Publicly available PacBio sequencing reads from *D. melanogaster* (Kim et al. 2014) were aligned to the heterochromatin-enriched *D. melanogaster* genome (Chang et al. 2019) with pbalign (SMRT v7.0), and IPDs were computed at nucleotide resolution with ipdSummary.py using the P5C3 chemistry (https://github.com/PacificBiosciences/kineticsTools/tree/master/kineticsTools). This outputs an IPD value which is an average of three IPD subheads values per nucleotide. All normalization of intermolecular variability and trimming for outliers was done automatically. Then, using the positive strand, all regions predicted to be Z-DNA by Gquad with a likelihood of two asterisks or higher were extracted in 300 base pair windows. The IPDs values of these sequences were extracted such that the predicted sequence to form Z-DNA was centered. All windows with no IPD values were filtered out, after which the IPD values of all sequences were averaged lengthwise and plotted.

### Gquad and SIST Cumulative Plots

For the SIST Cumulative plots, SIST results for melt, Z-DNA, and cruciforms were combined using element-wise maximums and plotted in Python. For Gquad cumulative plots, the number of asterisks for each type of non-B DNA were added for each base position and plotted in Python.

### Statistical Tests and Graphs

The two-sample Kolmogorov–Smirnov test was used to compare distributions of SIST and GQuad likelihood values. One-sample *t*-test was used for both the dyad density and G4Hunter distributions. Outliers were removed if they occurred more than 1.5 times the interquartile range away from the first and third quartile of the respective data.

Data were graphed using GraphPad.

## Supplementary Material

Supplementary data are available at *Genome Biology and Evolution* online.

## Supplementary Material

evac054_Supplementary_Data

## Data Availability

All data are available upon request. Genomic data used for analysis were published elsewhere and references are provided in the text. All code used is deposited on Github.
