# Supplementary material for: Enrichment of Non-B-Form DNA at D. melanogaster Centromeres
Source: Genome Biol Evol. 2022 Apr 20;14(5):evac054. doi: 10.1093/gbe/evac054 (PMC9070824; doi:10.1093/gbe/evac054)
Supplement: evac054_Supplementary_Data [file evac054_supplementary_data.zip › Supplemental Information Legends_211229.docx]

## Supplemental material

## Supplemental data

**Table S1. Table ranking the average Gquad value for all repeats in the *D. melanogaster* genome.** Repeats associated with centromere contigs are highlighted in yellow.

**Table S2. Table ranking all contigs that make up the genome based on the average Gquad likelihood.** Centromeric contigs are highlighted in yellow.

**Figure S1. Contributions of different non-B DNA types for each centromere.** Pie charts showing the percent of non-B DNA that is likely to be formed for each centromere as identified by Gquad. Asterisks indicate p<0.05 (One-sample t-test) compared to controls.

**Figure S2. SIST probabilities of adopting non-B DNA across centromeric contigs.** Cumulative plots showing the probability of non-B DNA formation for each base pair of each centromere contig at 25^o^C. The base-pair (kb) positions of the centromeric islands and flanking satellites are indicated below the plot and labeled in the legend.

**Figure S3. Gquad likelihoods of adopting non-B DNA across centromeric contigs.** Cumulative plots showing the likelihood of non-B DNA formation for each base pair of each centromere contig at 25^o^C. The positions base-pair (kb) of the centromeric islands and flanking satellites are indicated below the plot and labeled in the legend.
