# Supplementary figures and images for: Enrichment of Non-B-Form DNA at D. melanogaster Centromeres
Source: Genome Biol Evol. 2022 Apr 20;14(5):evac054. doi: 10.1093/gbe/evac054 (PMC9070824; doi:10.1093/gbe/evac054)

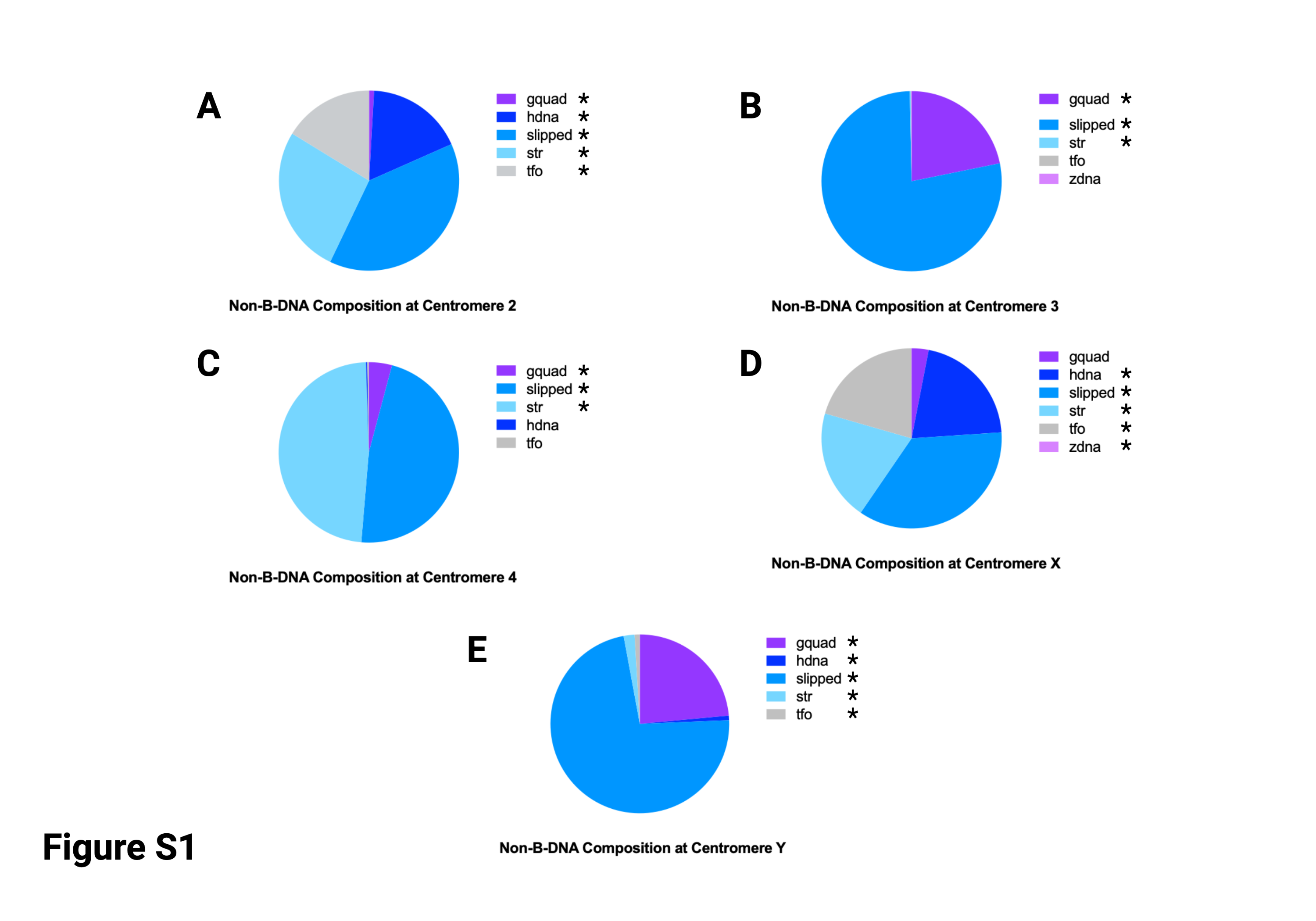

Supplement: evac054_Supplementary_Data [file evac054_supplementary_data.zip › Fig. S1 - Individual Distributions of Non-B-DNA at all Centromeres.png]

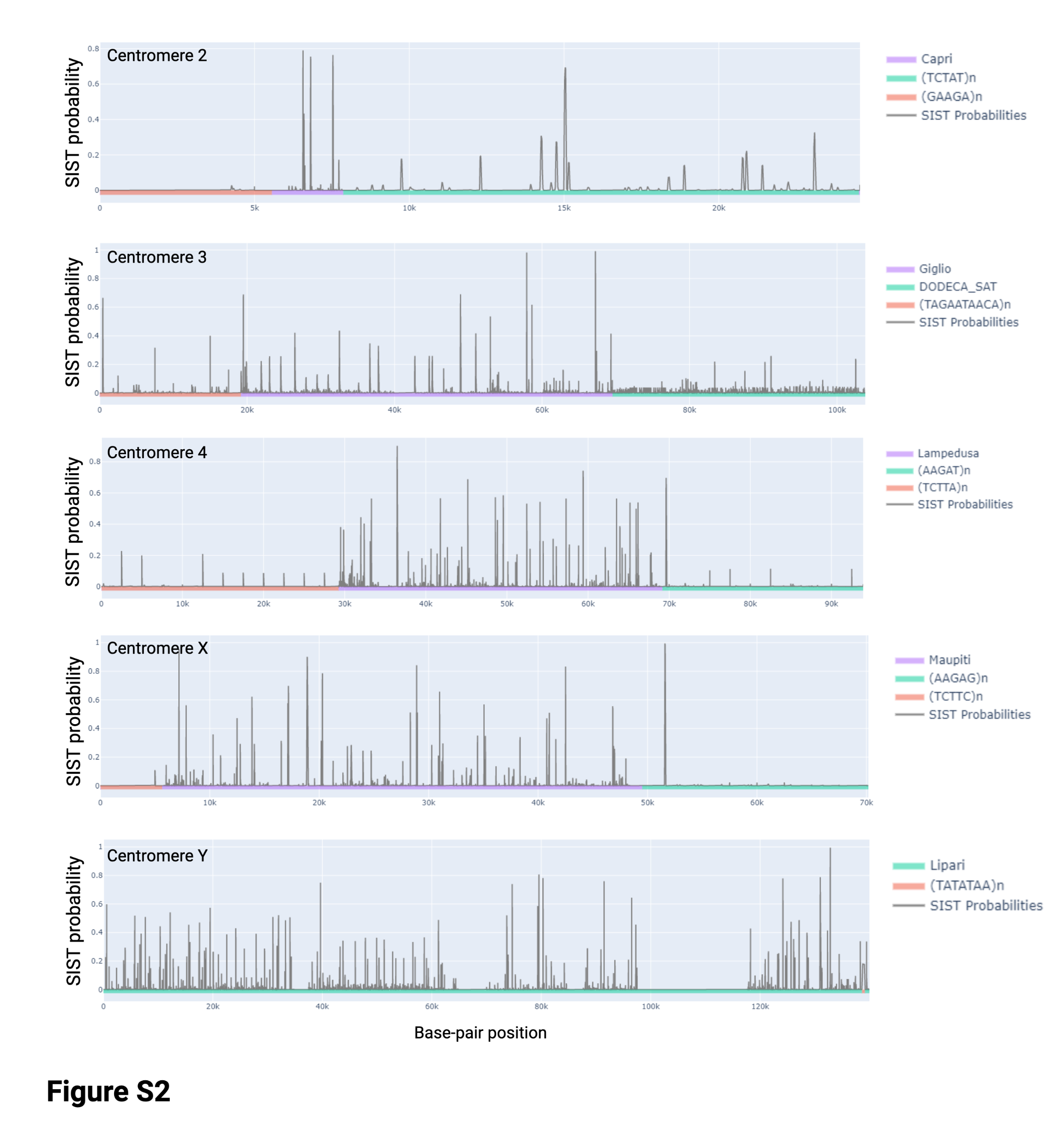

Supplement: evac054_Supplementary_Data [file evac054_supplementary_data.zip › Fig. S2- Cumulative SIST.png]

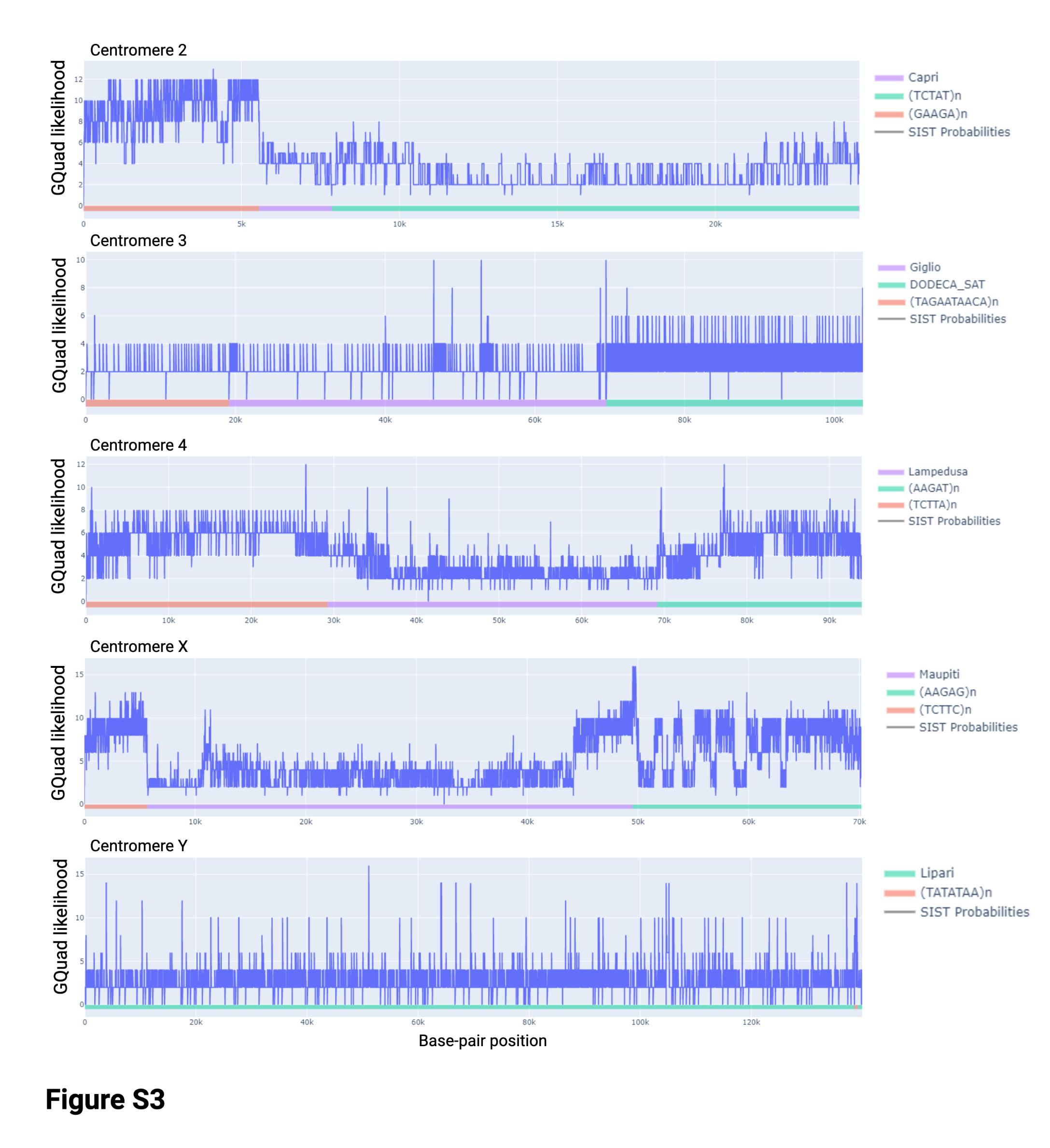

Supplement: evac054_Supplementary_Data [file evac054_supplementary_data.zip › Fig. S3- Cumulative GQuad.png]
